# Feasibility of Online High-Intensity Interval Training (HIIT) on Psychological Symptoms in Students in Lockdown During the COVID-19 Pandemic: A Randomized Controlled Trial

**DOI:** 10.3389/fpsyt.2022.904283

**Published:** 2022-06-21

**Authors:** Arnaud Philippot, Pauline Moulin, Marie-Hélène Charon, Costantino Balestra, Vincent Dubois, Philippe de Timary, Anne De Volder, Yannick Bleyenheuft, Kate Lambrechts

**Affiliations:** ^1^Institute of Neuroscience, Université catholique de Louvain, Brussels, Belgium; ^2^Psychiatric Hospital Area+/Epsylon ASBL, Brussels, Belgium; ^3^Environmental, Aging (Integrative) Physiology Laboratory, Haute Ecole Bruxelles-Brabant (HE2B), Brussels, Belgium; ^4^Department of Adult Psychiatry, Cliniques Universitaires Saint-Luc, Brussels, Belgium

**Keywords:** anxiety, depression, stress, High-Intensity Interval Training, exercise, students, lockdown, COVID-19

## Abstract

**Objective:**

We aimed to evaluate the feasibility of an online High-Intensity Interval Training (HIIT) program on clinical psychological symptoms in higher education students in the context of the COVID-19 pandemic lockdown.

**Materials and Methods:**

During the lockdown, 30 students aged 18–25 years, who had been screened previously with a cut-off score ≥5 in the Generalized Anxiety Disorder-7 (GAD-7) questionnaire, were randomly assigned to either the 4-week HIIT program with three sessions per week conducted through online videos, or a no-intervention control group. The primary outcome was the feasibility assessment. The secondary outcome was a psychological self-report with the 21-items Depression, Anxiety, and Stress Scale (DASS-21). Assessment and intervention were performed in compliance with social distancing rules.

**Results:**

Two participants in the HIIT were lost to follow-up, leaving 13 participants vs. 15 in the control group. We observed high adherence (87%) and complete safety for mental and physical status with the HIIT intervention delivered by online videos. The Mann-Whitney test demonstrated a significant (group × time, *P*-Value = 0.046) reduction of clinical stress symptoms and a trend (group × time, *P*-Value = 0.08) toward reduction of clinical depression symptoms, both favoring the HIIT group. No significant (group × time, *P*-Value = 0.118) interaction was found for anxiety symptoms.

**Conclusion:**

The online HIIT program was found to be feasible and safe in a clinical sample of young adults, who were experiencing social and physical restrictions due to COVID-19. HIIT reduced stress and depressive symptoms and thus these preliminary results show promise for broader application among higher education students during the present lockdown necessitated by the global COVID-19 health crisis.

## Introduction

The incidences of depression and anxiety symptoms doubled to 21% between 2018 and 2021 in the Belgian adult population, according to data collected during the lockdown necessitated by the COVID-19 pandemic (1). A recent meta-analysis and meta-regression including 226,638 individuals in 60 studies similarly showed a 24.0% global prevalence of depression and 21.3% for anxiety across the world during the COVID-19 pandemic (2); clinically significant symptoms were noted in association with risk factors such as female gender and youth (3, 4). Indeed, during the second lockdown in Belgium (March 2021), 38% of young people (18–29 years) suffered from depression, as evaluated by a Patient Health Questionnaire, and 34% suffered from anxiety, as evaluated by Generalized Anxiety Disorder-7 (GAD-7) (1), in accordance with surveys during COVID-19 waves in Western countries (5–8). Another recent meta-analysis for students in higher education in China (12 studies) and other countries (eight studies) revealed approximate doubling of the prevalences of depression (54%) or anxiety (37%) relative to corresponding results in surveys conducted prior to March, 2020 (4). Generally speaking, young people are apt to experience fears and uncertainties arising from their continued physical and social isolation during pursuit of a degree course. There is evidence for a causal association whereby anxiety precedes depression, such that many young people with depressive symptoms suffer from comorbid anxiety (9). The reduction of physical activity and sports due to constraints from the COVID-19 pandemic contributes to the stress experienced by students in higher education (10). Formerly active individuals who had been obliged to reduce their physical activity showed more symptoms of depression and loneliness, increased stress, and diminished ratings of positive mental health (11). On the other hand, individuals in the general population who managed to maintain their physical fitness in the face of the pandemic showed a 12–32% lower risk of experiencing anxiety and a 15–34% lower incidence of depression during the COVID-19 pandemic (12).

A recent meta-analysis compiling nine randomized control trials (RCTs), performed before the COVID-19 pandemic, showed a moderate improvement in state anxiety upon engaging in structured physical activity, as compared to no intervention or a minimal intervention control condition in groups of children and young people aged up to 25 years (13). However, there is a need for caution in interpretating such results, particularly with respect to establishing the dose-response relationship of different levels of intensity of exercise on anxiety symptoms in young people (13). Indeed, there is currently no consensus on the optimal frequency, intensity, type and duration of exercise to treat anxiety symptoms (13, 14). Moreover, High-Intensity Interval Training (HIIT) has gained increasing interest in recent years and has been ranked among the top fitness trends by the Association College of Sport Medicine (2022). In the adult population, HIIT has been shown to significantly improve cardiorespiratory fitness, cardiovascular function, anthropometric variables, exercise capacity, muscle structure and function (15). In addition, this type of training has been shown to reduce symptoms of anxiety and depression in non-clinical individuals (15) and depressed moods in people with severe mental illness (16) with better enhancements than moderate continuous fitness session (17).

In contrast, in young people, HIIT yielded mixed results in depression (18–20), anxiety and stress decreasing (18, 19, 21, 22). Despite the particular context of COVID-19, after restrictions in China, an encouraging RCT demonstrated depression reduction by running training supervised in person in a clinical sample in middle school (23). Concerning stress and anxiety, HIIT has raised questions about its impact on generating or reducing mechanisms (24). However, whereas the COVID-19 pandemic was a trigger for stress and anxiety in youth, home-based HIIT demonstrated a positive effect on symptoms of stress, anxiety in young people (21) and depression in adults (25), as well as showing a strong beneficial effect on moderate to severe anxiety symptoms pre-existing the pandemic (26). The inconsistency in results could be explained by the fact that the impact of HIIT has been overlooked on anxiety and stress disorders, as reported in a recent meta-analysis (16).

Therefore, given the deterioration of physical and psychological status observed in young people following the onset of the COVID-19 crisis, we sought to investigate the feasibility of an online HIIT intervention in a Belgian tertiary education student sample (aged 18–25 years) screened for clinical symptoms of anxiety during the lockdown arising from the COVID-19 pandemic. In addition, we also aimed to analyse preliminary findings on depression, anxiety, and stress symptoms after online HIIT. We hypothesized that the participants in the online HIIT would show better improvement in psychological symptoms compared to a passive control group.

## Materials and Methods

### Trial Design

We conducted a RCT to evaluate the feasibility of online HIIT intervention on psychological symptoms compared to a passive control group in tertiary education. The HIIT intervention entailed three sessions of 10 min per week and lasted for 4 weeks. Baseline and post-psychological testings were carried out the week before and after the 4-weeks follow-up. We followed the Consolidated Standards of Reporting Trials (CONSORT) checklist.

### Participants

Participants were recruited in a physical therapy higher education setting [Haute Ecole Bruxelles-Brabant (HE2B), Brussels, Belgium]. We offered them to participate in the study by contacting them through an email sent to all students in the month prior to the start of the study. We recorded descriptive characteristics and medication status of participants. Screening of general anxiety of participants was assessed with the GAD-7 (27). Written informed consent according to the Declaration of Helsinki and medical history was obtained from each participant. The study was approved by the Bio-Ethical Committee for Research and Higher Education, Brussels (No. B200-2020-088).

### COVID-19 Lockdown Conditions

Sciensano, the Belgian research center and national public health institute in charge of the pandemic statistics, reported that 21% of intensive care beds were occupied by COVID-19 patients in March 2021. The transmission rate was 1.02, and 288 of 100,000 Belgian citizens were infected on average per day in March 2021 (1). In response to the burgeoning caseload, Belgian government had decided to order closed the majority of non-essential shops, as well as sports clubs. The restrictive measures lasted between October 26, 2020 and June 9, 2021. A curfew was introduced from 10 p.m. to 6 a.m. in Brussels, the capital city.

### Eligibility Criteria

The inclusion criteria for participants were being a student in higher education, age between 18 and 25 years, having a score ≥5 in GAD-7, and accepting the principle of randomization in the trial. The exclusion criteria were refusal to participate, neurological history such as illness or head trauma, present severe and unstable respiratory disease, history of heart defect or cardiovascular disease or any other medical conditions prohibiting high-intensity sport or physical activity, substance abuse, i.e., alcohol or drugs, and being a high-level athlete. A comprehensive self-questionnaire was provided to students to collect all medical conditions to inform potential exclusion criteria and ensure safe participation.

### Randomization and Blinding

The week before and after the intervention, the students completed the DASS-21 questionnaire. Participants were anonymously assigned sequentially to one of two groups (HIIT and control group) using permuted block randomization with secret assignation. During the intervention sessions, therapists and participants were unaware of the individual scores used for randomization. The analysis was carried out in unlabelled group datasets.

### High-Intensity Interval Training Group

The HIIT intervention was conducted with session videos produced by a physical therapist (PM), which were available on an online platform for the participants. Table 1 displays two variants of sessions based on the main variables that can be manipulated to prescribe a HIIT session (28). Participants were free to decide when they wanted to perform their sessions during the week. We recommended having a rest day between sessions due to the high-intensity effort produced. The intervention consisted of 12 structured sessions of bodyweight intermittent aerobic and muscular strengthening exercises, designed to remedy a decline of aerobic fitness in lockdown (29). A block of three sessions to complete was sent to participants each week for 4 weeks, to a total of 12 sessions. Each session entailed alternating of High-Intensity Intervals (HIT) and active recovery intervals, each lasting 30 s to reach 10 min, see Table 1. The HIT comprised, for instance, jump squats, burpees, mountain climbers, jump forward lunge, jumping jack with a Rate of Perceived Exertion (RPE) of 6 or greater (1–10), following the modified Borg Analog Scale, which corresponded to a HRmax at least 80% (30). The active recovery intervals included plank, lateral plank, push-up, squat at a requested RPE of 4 or less. We encouraged to maintain the required intensity during each interval of the sessions. To promote motivation, each session was videotaped in different outdoor and indoor locations. All exercises employed own body weight for resistance, without use of special equipment. The HIIT sessions were carried out at the participants' home, thus in compliance with social contact regulations.

**Table 1:** Bodyweight HIIT circuit.

**Variant 1**	
Bear walk Back and forth squat jump Plank Ice skater Squat pulse Burpees Squat hold Back and forth lunge (left side) Superman breaststroke Back and forth lunge (right side)	Alternating between High intensity exercises (30 s) ≥6 RPE and active recovery exercises (30 s) ≤ 4 RPE
**Variant 2**	
Lateral lunge Run on the spot Back lunges Jumping jack Spider plank Diagonal knee kick, right hand/ankle touch Squat Diagonal knee kick, left hand/ankle touch Squat hold Floor touch jump squat	Alternating between High intensity exercises (30 s) ≥6 RPE and active recovery exercises (30 s) ≤ 4 RPE

*The 10-min session consisted circuit of 10 exercises (30 s each), repeated twice*.

Participants recorded their participation in the sessions in weekly reports sent to the trainer, who regularly guaranteed the monitoring for safe participation. These reports included attendance at the three sessions per week, their rate of perceived exertion, and possible questions about recovery, movement execution, and other issues addressed. The global RPE was assessed by the modified Borg Analog Scale (0–10) at the end of each session, as is standard in clinical studies (31). Participants were given a descriptive document indicating the corresponding intensity for each level between 1 and 10 (32), assuring validity and good reliability (30). Participants were physical therapy students and underwent fitness training during their course of study, ensuring proper execution of movements and knowledge on HIIT principles before starting the study.

### Control Group

Participants were requested to continue their usual activities without particular modification.

### Feasibility Primary Outcome

We evaluated the feasibility of the online HIIT intervention compared to a passive control group in the specific context of closure and distance measures imposed by the COVID-19 spread. We analyzed several outcomes: mean program adherence, individuals completed their sessions in a document sent each week to the supervisor; dropout rate per intervention; protocol monitoring with the collection of the mean RPE per session for each participant with the modified Borg scale (31); and safety concerning participants mental and physical progress with the psychological outcome and participant feedback.

### Psychological Secondary Outcome

The Depression, Anxiety, and Stress scales-−21 Items (DASS-21) (33) is a set of three self-reports. For each scale, there are seven questions (rated 0, 1, 2, and 3) related to depression, anxiety, and stress, thus providing three scores, each ranging from 0 to 21. The depression scale assesses dysphoria, hopelessness, devaluation of one's life, self-deprecation, lack of interest/involvement, anhedonia, and inertia. The anxiety scale assesses autonomic arousal, skeletal muscle tension, situational anxiety, and the subjective experience of anxious affect. The stress scale is sensitive to levels of chronic non-specific arousal, assessing difficulty in relaxing, nervous arousal, and being easily upset/agitated, irritable/over-reactive, and impatient. Scores for the three dimensions were calculated by summation, and the final severity score was obtained by multiplying by two. The DASS-21 has shown excellent internal consistency and temporal stability in large clinical samples (34).

### Sample Size

The sample size was calculated from a previous RCT, wherein we had evaluated Beck anxiety inventory scores after 2 weeks of physical exercise in tertiary education students suffering from clinical anxiety symptoms (35). To detect a decline in −8 points out of the maximum of 42 in the DASS-21 at the 5% significance level with a power of 85%, and assuming a standard deviation of seven points, we calculated a sample size of at least 15 participants per group.

### Statistical Analysis

The Mann–Whitney test was performed to determine the difference between the two groups over time regarding the outcomes for psychological scores. All statistical procedures were performed using the SPSS software (IBM SPSS Statistics 27.0, Armonk, NY, United States). The group identity of the data was anonymized for the statistical analysis. Results were reported along with their 95% confidence intervals (95% CI). A *P*-Value <0.05 was considered significant. The Empirical Rule Effect Size (ERES), defined at one half SD from the baseline score, was used to define clinically significant effect sizes of the treatment (36). A Pearson correlation analysis was performed on psychological symptoms between the baseline and difference scores (T1–T2) in the HIIT group to observe whether the level of symptom severity could be related to change over time.

## Results

### Study Sample

**Figure 1:**
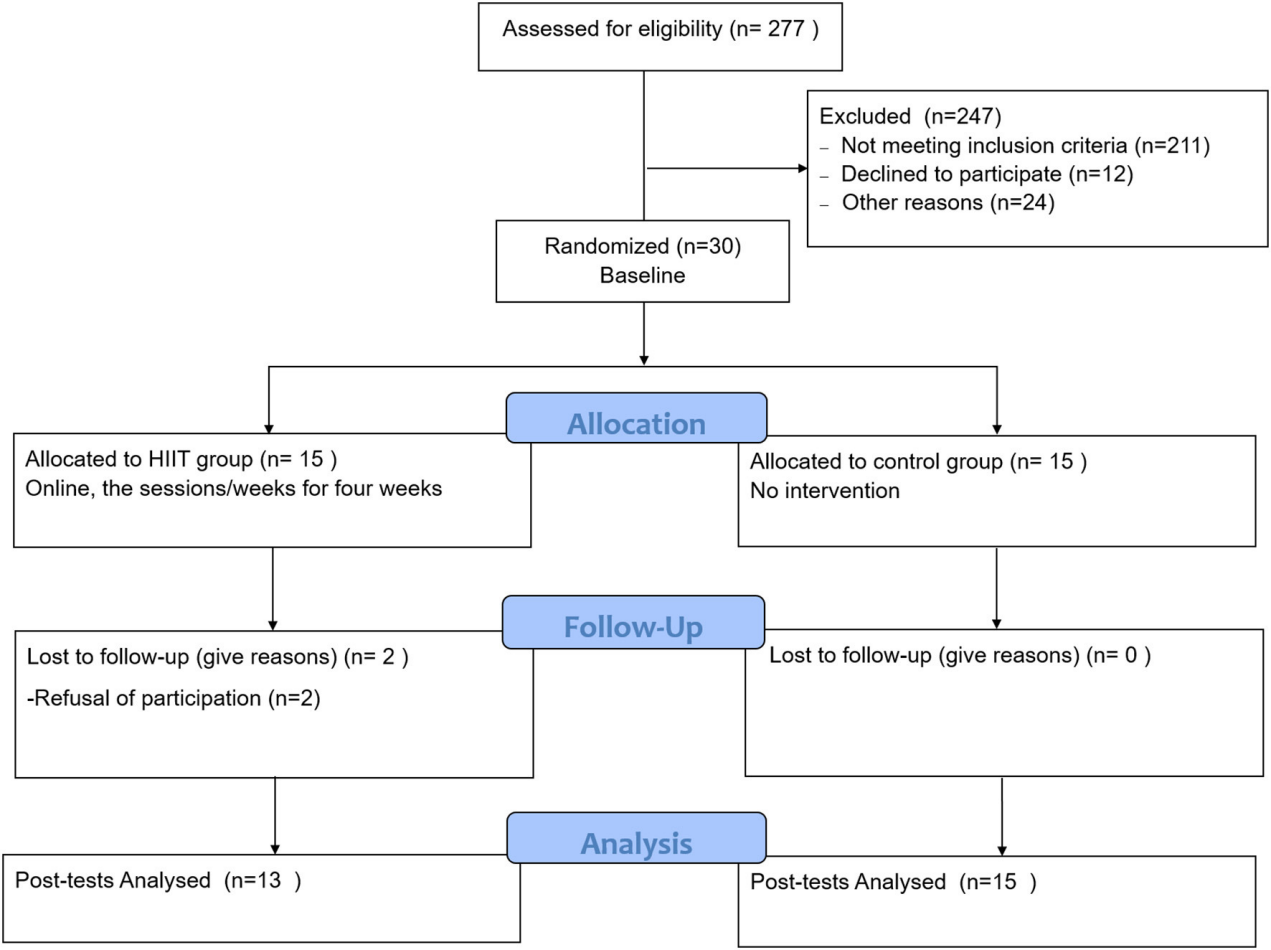
Consort flow-chart.

The experimental period ran from March 5, 2021 to April 2, 2021. Among the 30 students recruited at the baseline, two members of the HIIT were lost to follow-up (see Flow-chart, Figure 1 for details). The statistical analysis of pre-and post-test measures was thus restricted to the remaining 28 participants. The control group consisted of 14 women and one man of mean age 20.93 years (SD 1.94), whereas the final HIIT groups contained 11 women and two men of mean age 20.69 years (SD 1.44). For details of the study population, see Table 2. We calculated The Metabolic Equivalent of Task (MET) per hour to measure their physical activities following the updated compendium (37) and classified them per week following the recommendations from WHO (2021).

**Table 2:** Demographic characteristics of participants.

	**HIIT (*n* = 13)**	**Control (*n* = 15)**
**Mean age (SD)**	20.69 (1.44)	20.93 (1.94)
**Gender**		
Female	11	14
Male	2	1
Others	0	0
**Mean BMI (SD)**	22.67 (4.21)	21.35 (3.01)
**Living environment (Family, cohabitating, alone**)		
Alone	2	1
Cohabitating	6	7
Family	5	7
**Daily physical activities (MET.hour per week)** ^†^		
(<7.5 MET)	6	8
(7.5–15 MET)	5	4
(>15 MET)	2	3
**GAD severity**		
GAD 5–10 (mild)	5	7
GAD 11–15 (moderate)	5	5
GAD 16–21 (severe)	3	3
**SARS-2 infection** ^‡^	3	3
**Co-morbidities**		
ADHD	1	3
Eating behavior problems	1	0
Tobacco dependence	1	5
Physical disease	1	2
**Antidepressant or anxiolytic medication**	1	0
**Family history of mental illness (1st and 2nd degree relatives)**	6	4

*SD, standard deviation*.

† *7.5–15 Metabolic Equivalent of Task (MET) is recommended by the World Health Organization 2021*.

‡ *SARS-2 infection diagnosed before the study*.

### Feasibility Primary Outcome

In the HIIT group, the mean participation rate was 87%, and the dropout rate was assessed at 13.33%, with two participants lost because they refused to continue. The overall intensity set per session for each participant by the modified Borg scale (1–10) demonstrated a mean of 5.77 (SD 1.29), which is considered “hard” and a good execution of the intense effort provided by the participants. Regarding the safety assessment, the HIIT intervention did not reveal any increase in psychological levels, with the exception of one participant with a higher anxiety score of 2, increasing from 2 to 4 on 42-point scale anxiety of DASS-21. The control group yielded higher scores for seven participants, including five for anxiety, six for stress, and three for depression scales in the DASS-21. The students reported no adverse physical events, except for stiffness due to the heavy exertion.

### Psychological Secondary Outcome

**Figure 2:**
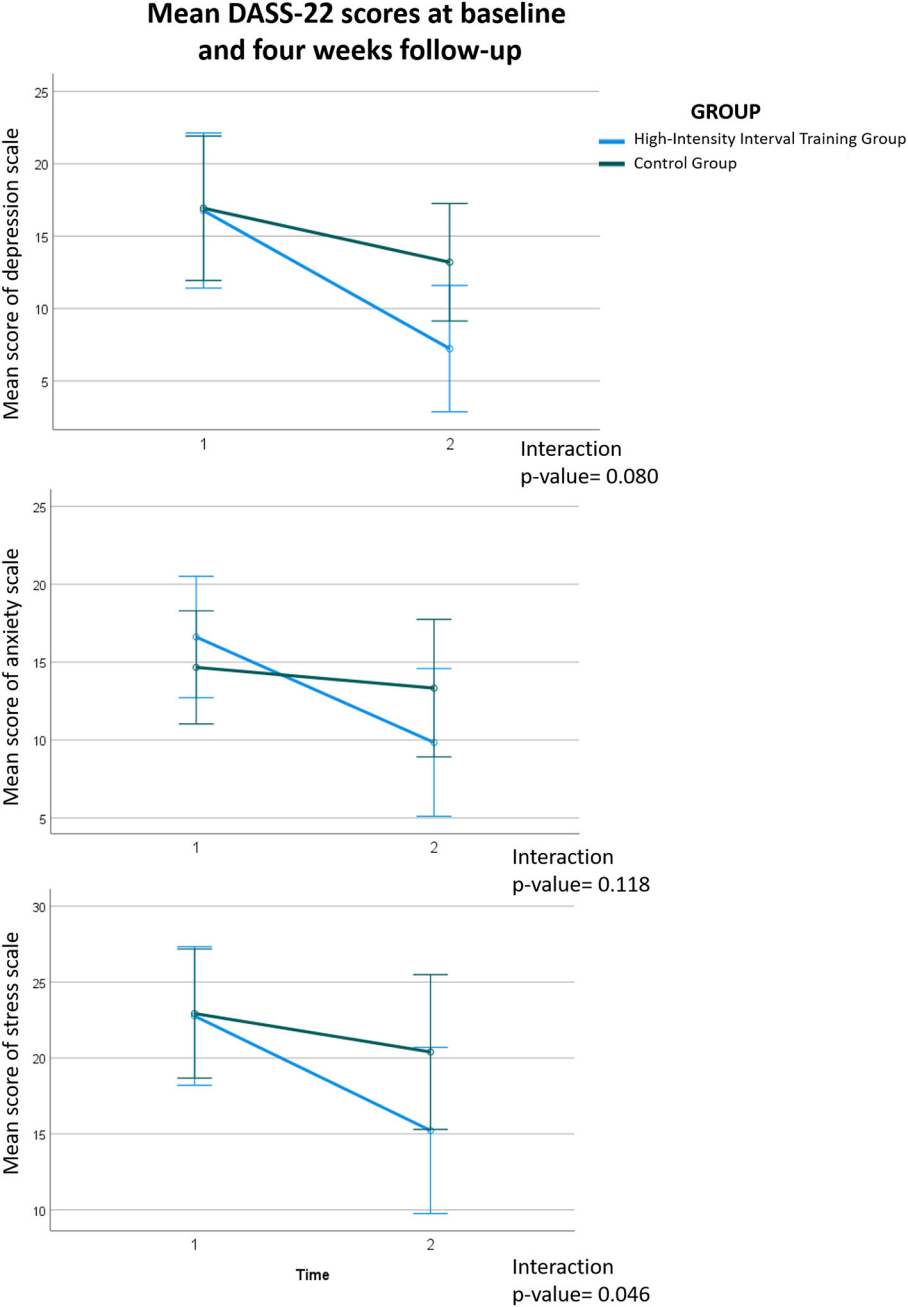
Graphs of depression, anxiety, and stress scales from DASS-21.

Scores and the level of severity according to the pathological thresholds at baseline and after intervention are presented in Table 3. In the stress scale of DASS-21, the Mann-Whitney test revealed a significant (group × change from baseline) interaction (*P* = 0.046) in favor of the HIIT group (Table 4 and Figure 2). After the intervention, students in the HIIT group had a mean decline (T1–T2) of 7.5 points on the 42-point scale [95% Confidence Interval (CI), 5.3–9.8] in their stress symptoms, progressing from a mean score indicating moderate stress symptoms, to a mean mild score. Students in the control group had a mean decline (T1–T2) of 2.5 points [95% (CI), −1.9 to 6.9] in their stress symptoms, this remaining on average at a moderate stress score. In the depression scale of DASS-21, Mann-Whitney test revealed a trend interaction (*P* = 0.080) in favor of the HIIT group (Table 4 and Figure 2). After the intervention, students in the HIIT group had a mean decline of 9.5 points [95% (CI), 5.4–13.7] in their DASS depression symptoms, thus progressing from a mean score of moderate depression to a mean normal score. Students in the control group had a mean decline (T1–T2) of 3.7 [95% (CI), −1 to 8.4] in their depressive symptoms, thus progressing on average from a score of moderate depression to one indicating mild depression. In contrast, the Mann-Whitney test showed no significant interaction (*P* = 0.118) in T1–T2 anxiety score changes between groups Table 4 and Figure 2).

**Table 3:** DASS-21 assessment.

**DASS-21**		**Baseline**						**Post-intervention**					
**Symptoms**		**Depression**		**Anxiety**		**Stress**		**Depression**		**Anxiety**		**Stress**	
**HIIT group (*****n*** **=** **13)**
mean (SD)		16.8	(9.6)	16.6	(7.2)	22.8	(8.7)	7.2	(6.6)	9.8	(7.5)	15.2	(8.3)
median (Min-Max)		16	[4-32]	18	[4-26]	22	[12-38]	4	(0–22)	8	[2-26]	14	[2-32]
**Severity**
Normal		4		2		3		9		6		8	
Mild		2		0		3		2		1		2	
Moderate		1		3		1		1		3		1	
Severe		6		8		6		1		3		2	
**Control group (*****n*** **=** **15)**
mean (SD)		16.9	(9.2)	14.7	(6.5)	22.9	(7.3)	13.2	(8.5)	13.3	(8.9)	20.4	(10.6)
median (min-max)		16	[4-32]	14	[6-26]	22	[10-34]	12	(0–26)	14	(0–34)	22	[6-42]
**Severity**
Normal		4		1		3		5		4		5	
Mild		1		3		2		3		0		1	
Moderate		5		5		3		4		4		4	
Severe		7		6		7		3		7		5	

**Table 4:** Results from the Mann–Whitney test on the difference (T1-T2) in intervention and control groups.

	**HIIT group**					**Control group**					**Interaction**
**DASS-21**	***n***	**Mean**	**(SEM)**	**[95%CI, lower to upper]**		***n***	**Mean**	**(SEM)**	**[95%CI, Lower to Upper]**		***P*** -Value^*^
Depression Symptoms	13	9.5	(2.1)	5.4	13.7	15	3.7	(2.4)	−1	8.4	0.80
Anxiety symptoms	13	6.8	(1.7)	3.5	10.0	15	1.3	(2.7)	−3.9	6.6	0.118
Stress symptoms	13	7.5	(1.1)	5.3	9.8	15	2.5	(2.2)	−1.9	6.9	0.046

* *Evaluated by Mann-Whitney test*.

A correlation analysis was performed to determine if a connection existed between the severity at the baseline and the reduction over time in psychological scores in the HIIT group. A significant and negative result was found for depression (*P*-Value = 0.004, *r* = −0.734, Pearson correlation), meaning that higher depression scores at baseline have a high potential to decrease. No significant result was shown for stress and anxiety (all *P*-values >0.05, Pearson correlation).

In the HIIT group, the mean score of improvement in symptoms of stress (7.5) exceeded the ERES score (4.4) and as did likewise the mean improvements in depression (9.5) compared to its corresponding ERES (4.8), demonstrating a clinically significant effect size of HIIT treatment. In the HIIT group, all 13 participants experienced a reduction in their stress scores, while only 8 of 15 (53%) of control participants experienced a reduction, vs. 6 of 15 (40%) who experienced a worsening, and one of 15 (7%) with unchanged stress.

## Discussion

We undertook a RCT to test the feasibility of participating in a brief, online HIIT program that could decrease scores of clinical anxiety, stress, and depressive symptoms compared to a passive control group from sample of students undertaking higher education, while screening with the GAD-7 for symptoms of anxiety, during a government mandated lockdown context during the COVID-19 pandemic. The HIIT intervention was considered feasible with high adherence (87%), acceptable dropout rate (13%), and safe intervention for physical and mental health. As predicted, HIIT group enjoyed a significant decrease in stress symptoms and a trend for reduction of depressive symptoms as compared to the control group. Contrary to expectations, we did not find that the intervention reduced the extent of anxiety experienced by the participants.

The HIIT intervention exhibited high participation, limited participant dropout rates, and good tracking of the intensity of effort produced during the sessions. Online training with short 10-min sessions, freedom to decide when to practice, and weekly supervision appeared to work well. Previous studies with an online exercise intervention in youth have also demonstrated good adherence and a correct dropout rate in adults with HIIT during confinement imposed by COVID-19 with 77.78 and 22% (25) and a moderate aerobic program in college students with 92.08 and 3.33% (38). Another online HIIT during the COVID-19 pandemic in youth revealed no dropouts but did not inform adherence (21). In contrast, a face-to-face HIIT session with university students resulted in lower average participation of 66.7%, likely due partly to the chosen schedule (8:15 a.m.) (39). The online mode with the freedom to decide when you want to train, as we performed, could be interesting to continue to be explored. HIIT did not produce any adverse effects for physical and mental health safety in the present study. Only one participant slightly increased his anxiety score, but it could be negligible due to his weak score under the cut-off. Moreover, the difference is striking when compared to the control group, where seven participants observed a deterioration in one or several psychological symptoms. Being a student during the COVID-19 pandemic, accentuated by a state of confinement, may expose these youth to more negative emotions (3, 4). As previously suggested, exercise may have a protective effect on mental health (40, 41). Our HIIT intervention may have revealed this protective effect during a major social crisis compared to the control group. Finally, our results are consistent with those of the face-to-face HIIT intervention, with average adherence rates greater than 80% and no acute injuries throughout the population, according to a meta-analysis of 33 systematic reviews (15). Attendance is crucial to achieving effective exercise treatment in mental health (40, 41). Our online HIIT might be a feasible intervention strategy for the prevention and treatment in mental health. Despite this promising result, our sample size is small, and further work is needed to establish the viability of online HIIT in a clinical sample of youth.

The robust reduction of depressive symptoms in the HIIT intervention group was in line with a previous systematic review and meta-analysis (42), and also in accordance with our recent RCT on the effect of physical activity in young people (aged 12–25 years) experiencing a present diagnosis or threshold symptoms of depression (43). Following the high and negative correlation (*r* = −0.734) between the severity of depression and its reduction in the HIIT group, this intervention may be considered as an efficient antidepressant for clinical symptoms. The antidepressant effect of HIIT in this present study could be explained by biological and psycho-social mechanisms (41). In the brief course of our 4 week program, we suppose that clinical improvement might reflect neuroplasticity pathways involving such factors as improved brain-wide vasculature function, a well as neuronal effects mediated by neurotrophin release. Downstream effects on neuroendocrine response, neuro inflammation, and oxidative stress may also contribute to the improved depression scores in the HIIT group (44, 45). Regarding psycho-social mechanisms, exercising participants might experience improved physical and body image self-perceptions. While brief in duration, completing our HIIT program is a difficult task calling for a considerable expenditure of effort. Thus, it may suffice to improve participants' skill mastery and lower their barriers against self-efficacy. HIIT may also simultaneously enhance the quality of sleep and depression and thus could be a good complement to current therapeutic modalities (46). The reductions in depression and perceived stress favoring the present HIIT group also seem in accord with two systematic reviews concluding that initial first positive effects of HIIT on mental health are mediated by acute improvements in cardiorespiratory fitness. However, those studies were conducted in older subject groups with history of psychiatric illness (15, 17). Previous tests of an HIIT program in younger subjects did not indicate significant changes in depression symptoms (20). This difference might reflect differences in participant samples or HIIT design or their context preceding the onset of the COVID-19 pandemic and associated restrictive social measures.

Unexpectedly, we did not find any effect of the HIIT on the extent of anxiety experienced by our participants. Present results are thus at odds with a recent meta-analysis showing that physical activity could help to alleviate anxiety symptoms in young people (13). However, that study did not address the clinically relevant anxiety considered in our study, nor did it consider specific effects of the pandemic on anxiety. Furthermore, the changes in anxiety in the present study, although not significant, showed a moderate effect size (Cohen's d = 0.8). Hence, the *P*-Value (*P* = 0.118) for the interaction does not exclude the possibility that a larger sample might have yielded significant results; given the variances in anxiety scores, we can estimate that a population size of 20 per group might suffice to detect effects of the intervention. An additional consideration is that our participants had at recruitment elevated scores of trait anxiety on the GAD-7 score, such that their specific anxious symptoms might have been more resistant to the intervention. Given the mixed results for RCTs against anxiety (19, 20, 25, 26, 38), we see a need for further investigation of the impact of HIIT on anxiety symptoms. A clinical sample screened for anxiety may show better improvements (21, 26). It seems that severity of anxiety, current fitness, exercise history, and exercise volume may be moderating factors for changes in cardiovascular and mental health after HIIT. Still, the mechanisms explaining their interactions are not clearly elucidated (14, 20).

The social isolation exacerbated by lockdown during the COVID-19 pandemic has detrimental psychological effects resembling those seen previously in diverse contexts such as space travel, polar or submarine expeditions, prison detention, certain military situations, or for patients in intensive care units (47). Therefore, we see prospects for a general utility of HIIT interventions in circumstances enforcing social isolation. The high adherence (87%) observed in our online HIIT intervention group indicates good tolerance, and may reflect its positive effects on wellbeing, notably with respect to reduction of depressive symptoms.

### Strengths and Limitations

We demonstrated a feasible trial with high adherence (87%) with the HIIT intervention delivered by online videos. Safety was assured for participants mental and physical health who practiced HIIT, with even a potential protective effect in mental health during the current social crisis. This is the first RCT demonstrating a positive impact of HIIT on psychological symptoms in a clinical sample of adult students in higher education. Furthermore, we tested the effect of the intervention in the context of lockdown during the COVID-19 crisis, which is a distinct stressor and risk factor for this population.

However, we must exercise caution in the interpretation of the preliminary results in our feasibility trial, given the small sample (*n* = 28), which may have been insufficient to detect effects on anxiety scores. We see scope for testing dose-effects of the intervention, as well as the long-term effects of the intervention on psychological scores. Due to circumstances of the COVID-19 pandemic, the HIIT providers were not present face-to-face during the sessions, which might have reduced the quality of follow-up, although we did supervise participation online. The participants were physiotherapy students, which could contribute to their strong adherence to the program. The HIIT group received more attention than the passive control group, which may be involved to some extent in the improvement in psychological symptoms. For generalization of present results, we need to investigate a broader group of participants from the general population.

### Conclusions

Our study could be considered feasible and safe. The preliminary observations supported our hypothesis that the HIIT intervention could reduce stress and with a tendency depression symptoms in a clinical sample of adult students engaged in tertiary higher education during a challenging lockdown period of the COVID-19 pandemic. The analysis was unable to demonstrate a significant impact on anxiety. Further studies are recommended to confirm and extend these positive effects of the HIIT program on the mental health of young people, notably in a broader population and with consideration of possible dose-effects of a longer HIIT program. In addition, the neurobiological, psycho-social, and behavioral mechanisms induced by HIIT need to be explored to understand better its effect on mental health in a clinical sample of youth.

## Data Availability Statement

The raw data supporting the conclusions of this article will be made available by the authors, without undue reservation.

## Ethics Statement

The studies involving human participants were reviewed and approved by the Bio-Ethical Committee for Research and Higher Education, Brussels (No. B200-2020-088). The patients/participants provided their written informed consent to participate in this study.

## Author Contributions

AP and PM contributed to the study design, were involved in the two arms of the trial at all steps, contributed to the statistical analysis, collected anamnestic data, and wrote the first draft of the manuscript. M-HC was involved in the two arms of the trial at all steps and in the collection of anamnestic data. AD, VD, PT, and CB gave comments and advice on interventions, study design, analysis, and draft manuscript. YB and KL conceptualized the project and oversaw its implementation, contributed to the study design, and wrote the manuscript. At the final step, all authors had access to the study data that support the publication. All authors contributed to the article and approved the submitted version.

## Funding

AP was funded by the Funds Baillet Latour in partnership with VD.

## Conflict of Interest

The authors declare that the research was conducted in the absence of any commercial or financial relationships that could be construed as a potential conflict of interest.

## Publisher's Note

All claims expressed in this article are solely those of the authors and do not necessarily represent those of their affiliated organizations, or those of the publisher, the editors and the reviewers. Any product that may be evaluated in this article, or claim that may be made by its manufacturer, is not guaranteed or endorsed by the publisher.

## Abbreviations

HIIT: High-Intensity Interval Training
GAD-7: Generalized Anxiety Disorder-7
DASS-21: Depression, Anxiety, and Stress Scale-21 Items.
